# Expected advances in human fertility treatments and their likely translational consequences

**DOI:** 10.1186/s12967-018-1525-4

**Published:** 2018-06-04

**Authors:** Norbert Gleicher

**Affiliations:** 1 The CHR, 21 East 69th Street, New York, NY 10021 USA; 2 The Foundation for Reproductive Medicine, New York, NY 10021 USA; 3 Laboratory for Stem Cell Biology and Molecular Embryology, Rockefeller University, New York, NY 10065 USA; 4 Department of Obstetrics and Gynecology, Vienna Medical School, 1090 Vienna, Austria

**Keywords:** Infertility, Gametes, Zygotes, Embryos, Cell lineage determination, Mosaicism, Maternal tolerance, Invasiveness, Implantation, Ovarian cycle

## Abstract

**Background:**

Due to rapid research progress in reproductive biology and reproductive clinical endocrinology, many human infertility treatments are close to potential breakthroughs and translational applications. We here review current barriers, where such breakthroughs will likely come from, what they will entail, and their potential clinical applications.

**Main text:**

The radical nature of change will primarily benefit older women, reduce fertility treatment costs and thereby expand access to treatment. A still widely overlooked prerequisite for implantation and normal pregnancy maintenance is timely development of maternal immunological tolerance toward an implanting paternal semi-allograft, if malfunctioning associated with implantation failure and pregnancy loss, while premature termination of tolerance appears associated with premature labor, pre-eclampsia/eclampsia and gestoses of pregnancy. Common denominators between pregnancy and invasive malignancies have again been attracting attention, suggesting that, like in malignant tumors, degrees of embryo aneuploidy may affect invasiveness and ability to “disarm” the immune system’s innate response against implanting embryos. Linking tolerance to implantation, we offer evidence that the so-called “implantation window” is likely immunological rather than hormonally defined.

**Conclusions:**

Because many here outlined treatment changes will disproportionally benefit older women, they will exert a pronounced effect on society, as increasing numbers of women at grandparental ages will become mothers.

## Background

For reproductive biologists and reproductive clinical endocrinologist these are exciting times—possibly the most exciting times ever! At many different fronts, both disciplines appear on the verge of groundbreaking breakthroughs with remarkable potential impacts on fertility treatments, prevention of genetic diseases but also on other areas of medicine.

We foresee women having genetic offspring into ages beyond menopause, chemo- and radiotherapies no longer being barriers to future genetic motherhood and paternity, and rather than disposed if genetically abnormal, embryos will be tested and, if abnormal, “repaired”, utilizing newly developed genetic editing techniques [1]. Successful in vitro maturation of primordial follicles appears very close; preliminary models of oocyte-producing artificial ovaries have already been reported [2]; normal oocytes and spermatozoa have been produced in mice by reprogramming somatic cells into induced pluripotent stem cells (iPSCs) and, then, into oocytes and spermatozoa. Generations of healthy pups were the result [3]. The same achievement in humans, is just a matter of time.

Many of these developments will expand women’s reproductive lifespans. Considering likely cost savings, expanding affordability and access to fertility services—all highly desirable developments, changes will be disruptive in how fertility services will be provided. With older women already the most rapidly growing age group having children [4], major societal adjustments must follow, affecting some of society’s most basic social and medical covenants: Schools will teach more children of older parents, and maternity services in hospitals will face more higher risk patients. Medical complications of pregnancies will not only be more common but, at times, also more severe [5]. In other words, society will have to adjust to a new generation of parents at what used to be grandparental ages.

Disruptive changes to the fertility industry, therefore, must be anticipated [6, 7], likely even exceeding the changes brought about by the 1978 introduction of in vitro fertilization (IVF) by Steptoe and Edwards [8]. IVF did radically revolutionize female as well as male fertility treatments, while with so-far almost seven million IVF births worldwide, also changing the world.

Even scientists and physicians initially viewed IVF, however, with a degree of suspicion—some even considered it ethically flawed. New potential accomplishments in human fertility treatments now face similar concerns: “Organoids,” recently defined as, *cells (that) have an intrinsic ability to self*-*assemble and self*-*organize into complex functional tissues and organs* [9], are increasingly used as experimental *in vitro* substitutes for human in vivo experiments. Because they mimic in vivo human organs and even human embryos (i.e., “embryoids”), they have become controversial, leading to demands that, for ethical reasons, limits be imposed on their utilization. Somewhat provocatively, some authors even renamed organoids *synthetic human entities with embryo*-*like features (SHEEFs)* [10]. Interspecies-chimeric experimentations have faced similar ethical concerns, despite their rather obvious importance for potential human organ-generations for transplantation purposes in other species [11, 12].

Other potential innovations are also under intense scrutiny [13], often held up by government regulations, guidelines issued by professional bodies or quasi government agencies [14] and/or national and international consensus agreements [7]. Though ethical concerns are especially warranted in experimentations with human reproduction, almost 40 years of IVF practice well demonstrated the respect clinical and research communities in reproductive biology and medicine have given to these concerns.

## Government regulation

IVF in the U.S. successfully evolved without government contributions. Already in 1973, years before birth of the first IVF offspring, future funding of research affecting embryos and embryonic tissue was disallowed by Congress, while permitting research in the private sector [15]. Except for mandating from IVF centers annual reports to the Center for Disease Control (CDC), the U.S. government mostly maintained a hands-off approach.

This changed in 2001 after attempts at cytoplasmic exchanges (cytoplasm from oocytes of young donors was injected into oocytes of older women) were reported attempting to improve IVF outcomes, when the Food and Drug Administration (FDA) declared regulatory authority over all embryo manipulations with potential effects on the human germline. A briefing document distributed to IVF on May 9, 2002 stated: *We advised practitioners that FDA has jurisdiction over the use of human cells that have received transferred genetic materials by means other than union of gamete nuclei*. (https://www.fda.gov/ohrms/dockets/ac/02/ briefing/3855b1_01.pdf), thereby from this moment requiring Investigational New Drug (IND) exemptions for all such studies. Since INDs are the process required for new drug approvals, this announcement, instantly, made further research in this arena unaffordable for IVF centers.

Since then, things got even worse: By, through a provision of the *Consolidated Appropriation Act*, outright prohibiting the FDA from even reviewing proposals for INFs, Congress on December 18, 2015 reinserted itself even further [16]. Existence of this moratorium is extremely troublesome because it prevents in this area all clinical research and practice in the U.S.

Neither FDA nor Congress interposed themselves in such fashion during the initial clinical evolution of IVF. As long as IVF was considered “experimental,” supervision of clinical IVF programs by local Institutional Review Boards (IRBs) was considered adequate. Why such a framework is no longer considered appropriate now is unclear.

This moratorium currently prevents clinical trials of all cytoplasmic exchange procedures, whether for prevention of transmission of mitochondrial diseases from mothers or for fertility purposes, and also applies to Crispr-Cas9 [17] and similar gene-editing procedures in potential clinical applications to treat infertility. This was recently reaffirmed [18], when a New York IVF center performed spindle cell transfers utilizing donor cytoplasm [19] in preventing transmission of a mitochondrial disease from mother to offspring. By moving the embryo transfer to Mexico, the investigators attempted to circumvent the FDA’s regulatory authority. Considering that the embryo manipulation took place in a New York City, the FDA, foreseeably [20], followed up with a “cease and desist” letter [21].

Ultimately rewarded by a Nobel prize [22], worldwide success of IVF offers a good example, how reproductive clinical and science communities can responsibly manage ethically controversial issues with dignity and self-control without interference from government. Considering the overwhelming importance of human embryology research for stem cell sciences, regenerative medicine, immunology and oncology, at stake is no less than the country’s medical leadership in the world. The U.S. will have to find ways to allow responsible research to continue.

## Gametes

Oocytes and spermatozoa at fertilization contribute (in haploid format) their nuclear genomes (nDNA), while oocytes also contribute the females’ mitochondrial genome (mDNA). This explains why mitochondrial diseases, caused by mutations of mDNA, are only passed on by mothers [23].

Dogma still holds that women are born with all their follicles. Ovarian reserve (OR, describing quantity of remaining follicles/oocytes) constantly declines, starting in utero [24]. By age 51 (average time of menopause), follicles are in the low hundreds, and no longer respond to gonadotropins. By producing fresh spermatozoa into very advance ages, males, in contrast, maintain fertility.

In 2000, Xie and Spradling reported the existence of a germline stem cell (GSC) niche in ovaries of *Drosophila* [25]. Niches are regulatory microenvironments for stem cells, created by stromal cells. Here, three somatic cells acted as a niche, able to replace GSCs, lost by normal or induced differentiation.

These observations encouraged the hunt for GSC niches in other species, including humans. Almost two decades later, GSCs are well characterized in non-mammalian models, but their existence in mammals has remained contested [26–28]. With stem cells in other human organs defined, lack of evidence for human GSCs in ovaries is puzzling and inconsistent with current understanding of ovarian ontogeny. Their established existence in non-mammalian animal models further suggests that they, simply, have not been properly identified yet.

This may have different causes: GSCs may be uncommon and, therefore, difficult to isolate; currently known markers may not identify them satisfactorily; or GSCs may be identifiable by markers only while functionally active, which they only rarely may be. Though existence of human GSCs, therefore, appears likely, they do not necessarily play a significant role in the reconstituting OR.

In 2003, Hübner et al. generated in culture oogonia from mouse embryonic stem cells (ESCs) that entered meiosis, recruited adjacent cells to form follicle-like structures containing oocytes, which could be fertilized and cultured to blastocyst-stage [29]. In 2013 Hayashi and Saitou reported generation of oocytes from mouse ESCs and induced pluripotent stem cells (iPSCs) [30]. These experiments culminated in 2016 in a remarkable report by Hikabe et al. in which the authors reconstituted in vitro the entire cycle of the female germ line in the mouse, in the process reconstituting oogenesis from iPSCs, achieving fertilization and confirming downstream multigenerational healthy progeny [3]. In 2017 Hayashi et al. updated the methodology for reconstituting from iPSCs mouse oogenesis by shortening the process to only approximately 5 weeks [31]. Ishikura et al. in parallel reported derivation and propagation of spermatogonial stem cell activity in the mouse from iPSCs [32].

Similar experiments in humans, undoubtedly, are already underway. Once human primordial follicles can be cultured to maturity in vitro, a small ovarian cortical biopsy at young age, yielding a few hundred primordial-stage follicles, could, thus, virtually guarantee lifelong fertility into advanced ages. iPSCs, reprogrammed from peripheral skin cells, fibroblast, hair bulbs or other autologous somatic cells, then directed toward autologous oocytes and/or spermatozoa, would offer unlimited availability of gametes even without need for ovarian or testicular biopsies.

Unlimited oocyte and sperm gluts will be highly disruptive [7]. Developments most patients would greatly welcome, like women conceiving into much older ages, and obsolescence of ovarian stimulations via daily self-injections of gonadotropins will make fertility treatments more “patient-friendly” but will also greatly disrupt the pharma industry. Avoidance of the ovarian hyperstimulation syndrome (OHSS), fortunately a rare complication of ovarian hyperstimulation, can be viewed as improvement in safety of treatments [33].

Combining the use of iPSCs with the concept of repairing embryos with genetic defects (for more on this, see next section), Hirota et al. demonstrated that reprogramming of stem cells from trisomic mice into iPSCs, trisomic cells returned to normal haploidy in a process the authors named trisomy-based chromosome loss (TCL). They then differentiated these now normal iPSCs into the male germ line and functional sperm, producing chromosomally normal fertile offspring [34]. Though performed in sterile XXY and XYY mice, the authors pointed out that the technique was applicable to all trisomies, including Down’s syndrome (Trisomy-21), the most frequent viable trisomy in humans, opening intriguing possibilities for human treatments.

Their finding has also relevance for preimplantation genetic screening (PGS), recently renamed preimplantation genetic testing for aneuploidy (PGT-A) [35, 36], and further addressed below.

Until very recently, any aneuploidy detected in embryos through PGS/PGT-A led to embryo disposal. In July of 2016, transfer guidelines were, however, radically revised, now selectively permitting some transfers [37].

The reasons are clinical observations following transfers of presumed aneuploid/mosaic embryos (discussed later) and stem cell data: For example, embryos with abnormal PGS/PGT-A results can be source of euploid stem cell lines [38, 39]. In reverse, normal human iPSCs also exhibit pervasive mosaic aneuploidies [40]. Mouse experiments offered further evidence for the plasticity of early-stage embryos by demonstrating chromosomal self-correction downstream from blastocyst-stage more profoundly in the inner cell mass (ICM) than in trophectoderm (TE) [41]. Hirota et al. further defined this plasticity as a two-way street [34], and one is left wondering about the purpose of ploidy determinations at blastocyst-stage If embryos can self-correct further downstream [35, 36].

## Zygotes

The single-cell organism formed at fertilization from union of oocyte and sperm is a zygote. Its DNA is the combinations of both gametes’ genetic information, with a haploid secondary oocyte and a haploid male gamete unifying into a diploid cell. With the sperm entering the oocyte, the 2nd meiosis is completed. The result is a haploid maternal cell with half of its previous chromosomes (n = 23, nDNA), almost all of the oocytes original cytoplasm (including maternal mitochondria and, therefore mDNA), a haploid set of male chromosomes (n = 23, nDNA) as male pronucleus, and an extruded second polar body with another set of 23 of its chromosomes. The nDNA of female and male pronuclei then replicates, temporarily creating a quadroploid (n = 23 × 4 chromosomes) cell, thereby providing the substrate for fusion of the two pronuclei ca. 30 h post-fertilization, and the 1st mitotic division of the zygote into two diploid blastomeres with 23 × 2 chromosomes each.

Except during early days of IVF, when embryology laboratories were not yet able to culture embryos well in vitro, the zygote attracted limited attention in assisted reproduction [42]. As embryology improved, culture to cleavage-stage (day-3 after fertilization) became routine [43]—until blastocyst-stage transfer was proposed by Gardner et al. in the late 1990s [44].

A brief episode of secondary attention was awarded to the zygote when, initially through 1st polar body biopsy and later through 1st and 2nd polar body biopsies combined [45], Verlinsky et al. proposed the concept of PGS. Polar body biopsy was, however, manually too complex. Like embryo transfers before, PGS, therefore, quickly migrated to cleavage and later to blastocyst-stage.

The zygote recently, however, attracted renewed attention when Ma et al. reported improved success and accuracy with Crispr-Cas9-editing in correcting a dominant mutation in human embryos causing hypertrophic cardiomyopathy. They attributed their technical progress primarily to concomitant intracytoplasmic sperm injection (ICSI) of oocytes and injections of Crispr-Cas9 [6]. How the defective gene was, however, corrected in the process was questioned, and their results have so-far not been duplicated. If confirmed the zygote, however, may assume once again an important role in future gene-editing.

Similar to DNA editing with Crispr-Cas9, “base-editing,” may, at times, offer further advantages: Here, rather than short strands of DNA, single base mutations of RNA are “corrected” [46], and message rather than germline DNA is “edited.” Interventions, therefore, are temporary, and do not affect the germline by establishing permanent multigenerational changes in DNA. “Base-editing,” thus, offers potential new therapeutic opportunities for genetic editing, though with fewer short- as well as long-term risks, including undesired permanent mosaicism at offsite targets. Potential risks with Crispr-Cas9 and base-editing, including off-target effects, however, do warrant caution before such techniques are applied upon human embryos in a clinical practice setting.

Through preimplantation genetic diagnosis (PGD), current clinical practice allows diagnoses of hundreds of disease-causing single mutations in human embryos. Affected embryos are then routinely discarded, a still controversial practice, by some considered *reproductive discrimination* [47]. Using above noted genetic editing techniques, abnormal embryos should become “repairable” and, therefore, transferrable. This would add to size of available embryo pools for transfer and, therefore, improve pregnancy chances. Even current religious and ethical opponents of IVF, should view avoidance of disposal of embryos positively.

## Embryos

Cleavage-stage embryo transfers on day-3 after fertilization (6–8 cell stage) replaced pronuclear (zygote) transfers once embryology laboratories improved embryo culture conditions. By 1998, Gardner et al. however, claimed blastocyst-stage transfers (on days-5/6 after fertilization) to improve clinical pregnancy rates and reduce need for transfer of multiple embryos [44], thereby minimizing twin births. Though follow up studies did not confirm claims of improved clinical pregnancy rates in unselected patients, later studies confirmed marginally beneficial impacts on live birth rates, though only in good-prognosis patients [48]. Universal blastocyst-stage culture has, nevertheless, been gaining popularity ever since.

Best human embryos reach blastocyst-stage by day-5 after fertilization, and rarely by day-6. Sporadic pregnancies have even been reported from day-7 blastocysts. Two recent human in vitro implantation studies [49, 50], demonstrated, without need for maternal contributions, normal human embryo development up to day 14 after fertilization. Human embryos, thus, appear self-regulated far beyond implantation. Experiments were only terminated because international conventions currently still prohibit in vitro cultures of human embryos beyond day-14. For how long human embryos can survive and develop without maternal contributions is, therefore, still unknown.

Beyond current borders of viability (~ 22–24 weeks gestational age), abilities to successfully maintain extremely preterm infants are still poor. The par between already demonstrated embryo culture abilities and potential neonatal clinical viability, therefore, is only approximately 20–22 weeks. Using an ex vivo uterine environment, recent studies in very premature lambs demonstrated normal growth over a full week [51]. “In vitro *pregnancy*” (*IVP*), starting with IVF, and including long-term in vitro laboratory culture of embryos, followed by maintenance until potential viability in an ex vivo system, therefore, appears increasingly less utopian. Such an option appears also increasingly relevant, as women with absent uteri are increasingly exposed to risky and very costly uterine transplants [52].

We in this section, however, want to concentrate on two areas of research with considerable potential importance for the biological understanding of preimplantation-stage embryos—cell lineage determinations and embryo mosaicism.

## Cell lineage determination

The ability of one cell to give rise to all cell lineages (i.e., mesoderm, endoderm, ectoderm and germ cells) defines pluripotency. In the human embryo, cells that have this quality are a transient population, making up part of the so-called epiblast (i.e., ICM) which, ultimately, forms the embryo/fetus/offspring. The early preimplantation-stage embryo is made up of three distinctly different cell lineages—the embryonic epiblast, the extra-embryonic primitive endoderm and the TE, which ultimately forms the placenta.

Though differences between human and mouse embryo development are coming into focus [53], the mouse is still the principal subject of research [recently reviewed, 54, 55]. The importance of studies in human embryos was, however, recently reemphasized when OCT4 was demonstrated to play distinctively different roles in mice and humans [56].

Like human embryos, mouse embryos at cleavage stage are characterized by seemingly “equivalent” blastomeres. How these cells become either ICM or TE has remained controversial. To find the answer is important for a better understanding of early stages of human embryology—but has also clinical relevance for the increasingly popular PGS/PGT-A procedure in association with clinical IVF. Here either 1–2 blastomeres (at cleavage-stage) or 5–7 TE cells (at blastocyst stage) are biopsied to determine whether embryos are euploid and, therefore, transferrable or should be disposed.

Under assumption of ICM and/or TE lineage-dependent biases, a TE-biopsy should more reliably reflect the ICM if assignments of initial blastomeres to cell lineages are not biased but at random. Biased selection would, however, strongly suggest that TE biopsies cannot reliably reflect ICMs. Cell-fate biases, indeed, appear initiated as early as in the 2-cell stage and gaining pace at the 4-cell stage. They derive from methylation of arginine 26 on histone 3 (H3R26), which determines length of binding of important transcription factors to DNA. Longer binding fosters expression of Sox21 (and other genes) and drives cells toward the embryonic (ICM) lineage, while cells with shorter exposure will develop toward the TE (placental) lineage [57].

Most mouse studies reached the conclusion that such biases, indeed, exist [55, 58–60]. Niakan’s lab, however, recently again demonstrated that early embryo development distinctively varies in mice and men: By targeting and eliminating with Crispr-Cas9 in human zygotes the gene that encodes OCT4 (*POU5F1*), embryo development to blastocyst stage was compromised. In *POU5F1*-null-cells, gene expression was then found downregulated for extra-embryonic TE genes (i.e., *CDX2*) as well as for regulators of the pluripotent epiblast (i.e., *NANOG*). In the mouse, elimination of *pou5f1*, however, did not prevent blastocyst formation, though maintenance was impaired [56]. The importance of OCT4 for human blastocyst-stage development was recently also confirmed by Zernicka—Goetz’s laboratory [61].

As embryos transit from pre-implantation to post-implantation stages, pluripotency within the epiblast declines (in the mouse, for a considerable time period during gestation, it does not completely disappear), as Fibroblast Growth Factor (FGF), Bone Morphogenic Protein (BMP) and other agents affect differentiation of epiblast cells into specialized and developmentally restricted fates. Rapidly evolving knowledge surrounding cell lineage determinations, will have crucially important consequences for establishing fate-specific stem cell lines at specific developmental stages [54, 55] and, with it, for successful manipulations and reprogramming efforts of cell populations, for establishing organoids and similar cell-constructs for research [9, 62] and in treatments of genetic diseases, cancers, as well as in regenerative medicine.

## Mosaicism

How common mosaicism is in human preimplantation embryos has remained controversial. Mosaicism is defined as presence of more than once chromosomal cell lineage in a tissue, organ or embryo/individual. Because of a process called “microchimerism,” most, if not all humans, are chimeras, as during intrauterine life mosaic clones are routinely transmitted from mothers to offspring and vice versa [63].

Because of its alleged ability to improve outcomes, utilization of PGS/PGT-A in association with IVF has been increasing. Three consecutive generations of the procedure so-far have, however, been unable to demonstrate promised outcome benefits. We [35, 36] and others [64] have argued that significant underestimates of TE-mosaicism have been a principal reason, resulting in large numbers of false-positive diagnoses [64] and wasteful disposal of transferrable embryos. Especially in poorer prognosis patients with only small embryo numbers, erroneous disposal of healthy embryos will actually negatively affect outcomes.

TE-mosaicism in blastocyst-stage embryos may, indeed, be almost universal, and fulfill important physiological functions. In malignant tumors, degrees of aneuploidy correlate with invasiveness and obfuscation of patients’ immune responses to tumors [65]. Aneuploid/mosaic cell clones in TE, therefore, may be supportive of the invasive implantation process [66].

Additional observations have contributed to increasing skepticism about the efficacy of PGS/PGT-A. As currently practiced, PGS/PGT-A involves TE-biopsies at blastocyst-stage. Since TE represents the placental cell lineage, the procedure assumes that biopsies of the placental precursor structure offer reliable chromosomal information about epiblast (ICM), the fetal precursor representative of all three future germ layers and germ cells.

Discrepancies between TE and ICM in human embryos have, however, been reported [67]. In TE, but especially profoundly in the ICM, embryos also demonstrate remarkable plasticity capable of eliminating aneuploid cell clones downstream from blastocyst stages [41]. Though so-far directly only demonstrated in mice, evidence for similar human plasticity can be deducted: Embryos reported as aneuploid/mosaic by PGS/PGT-A have given rise to normal euploid stem cell lines [38, 39], while, at the opposite extreme, stem cells from trisomic mice returned to a haploid state when reprogrammed into iPSCs, [34]. Surprisingly excellent implantation, clinical pregnancy and live birth rates after transfer of embryos, by PGS/PGT-A reported to be aneuploid/mosaic [68–72], offer, however, the single most convincing evidence for such plasticity, and will, undoubtedly, lead to significant changes in embryology practice in IVF.

## Tolerance

Implanting embryos are paternal semi-allografts. The maternal immune system, therefore, should reject them; yet, in normal pregnancies competent maternal immune systems do not reject implanting embryos. They, therefore, must reprogram themselves from rejection to tolerance. Timely development of maternal tolerance, therefore, must be viewed as an absolute prerequisite for successful implantation and pregnancy maintenance.

Induction of maternal tolerance differs from induction of tolerance in solid organ recipients, where allogeneic antigen load is consistent, while in pregnancy it grows exponentially with advancing gestation. Also, organ transplantation requires permanent tolerance, while in pregnancy tolerance is only temporary (on average 40 weeks from last menstrual period).

Whether premature or at term, labor is increasingly considered caused by termination of this temporary tolerance [66]. Appropriate maternal tolerance levels, therefore, appear essential from implantation until labor. What produces the remarkable biological characteristics of maternal tolerance toward the paternal semi-allograft is, however, still largely unknown and is currently actively pursued in a number of research laboratories around the world.

Paradoxically, research efforts have primarily concentrated on local immune responses within the complex micro-environments of implantation sites [73]. Yet, since implantation sites *never* demonstrate evidence of allogeneic immune responses (even if implantation occurs extra-uterine), adequate tolerance levels must exist even before implantation occurs. Though some of the presumed steps in tolerance induction we here describe are still unproven, basic knowledge of how the immune system functions, supports a cascade of events, as here described.

What happens (in the micro- environment of implantation sites) immediately after implantation must, therefore, already represent a second stage in development of maternal tolerance. In absence of a yet undescribed first step in tolerance induction, implantation would either never happen or evidence of an allogeneic immune response must be visible. Women who lack this first stage tolerance level, likely, indeed, either do not implant (i.e., suffer from implantation failure) or miscarry so early that pregnancy is either not recognized or cannot be histologically assessed (i.e., chemical pregnancies).

For decades investigators have hypothesized that abnormal maternal immune function may be responsible for implantation failures [74] and pregnancy losses [75]. Presumed abnormal immune responses were, however, considered to be *autoimmune*—thus mistakenly leading to treatment of presumed *autoimmune*- rather than *alloimmune* responses [66], while failing to recognize that insufficient initial tolerance development was the real initial culprit causing maternal allogeneic responses.

In microenvironments around implantation sites, local immune responses also play important roles as part of a cascade of sequential tolerance-inducing layers that offer timely tolerance and the necessary redundancies that characterize all essential biological processes. Systemic tolerance pathways, likely induced during the 48 h immediately preceding implantation are, however, probably most essential for early implantation. Extra-uterine pregnancies, which in extreme cases can go to term [76], are the most convincing “natural experiment” in support of this assumption since they demonstrate convincingly that adequate tolerance levels needed for implantation evolve even in absence of uteri.

Once embryos invade the maternal host (whether via the endometrium or at extra-uterine sites), systemic tolerance pathways are then, likely, further augmented by local immune responses within the invasive microenvironments surrounding the implanting embryos. Facing a rapidly growing fetus and placenta and the challenges of a logarithmically growing antigenic mass, tolerance is, likely, further augmented by maternal and fetal microchimerism [63]. Starzl was the first to demonstrate that mutual microchimerism in donor organ and organ recipient was important for successful allogeneic solid organ transplantation [77]. It, likely, is equally important in establishing temporary tolerance in human pregnancy.

A desensitizing effect of semen on the maternal immune system has been suspected for decades since prior exposure to semen appears inversely associated with preeclampsia/eclampsia risk, [78, 79]. Since pregnancy chances in virgins are not reduced, semen exposure, alone can, however, not be presumed to induce sufficient tolerance. Like other essential processes in human reproduction, development of adequate tolerance, likely, depends on multiple inductive processes, which not only work in sequence but also serve as biological back-up systems, in cases one fails.

Embryos spend approximately 48 h “floating” within the microenvironment of the uterine cavity before implanting. This time period must have functional importance and can be assumed to allow communications between embryos and maternal immune systems. Investigations of secretory products during this time period, or in previously mentioned in vitro implantation models [49, 50] may, therefore, be revealing.

Timely induction of tolerance pathways, then allows implanting embryos to invade the endometrium (or, in cases of extra-uterine pregnancies, other tissues). Existence of such pathways can be deduced from another experiment of nature: Immune responses to selected helminths can positively or negatively affect female fecundity [80]. Like the fetal semi-allograft, helminths are parasites, their survival depending on adequate tolerance levels of the host. Some helminths appear to induce similar tolerance pathways to those induced by embryos. Women infected with those parasites, therefore, demonstrated improved fecundity.

Based on these observations, we predict the discovery of treatments that will induce tolerance-inducing pathways, which will decrease implantation failures and miscarriage rates and significantly reduce the incidence of premature labor especially in women with inflammation and autoimmune diseases, where prematurity is an almost universal phenomenon [81].

## Invasiveness

Like malignant tumors, implanting embryos possess invasive properties, and in both clinical circumstances a seemingly normally functioning immune system is circumvented. Except in malignant gestational tumors, embryos, however, quickly lose their invasiveness, while malignancies not only retain but, often, accelerate their invasive capabilities over time. Discovering the turn-off switch for time-limited invasiveness in implanting embryos, therefore, could have major relevance for cancer therapeutics.

Analogies between cancer and embryo invasiveness go further: Aneuploidy is a hallmark of invading tumors, found in 90% of solid tumors [82], a percentage quite similar to what now is considered mosaic aneuploidy in TE at blastocyst-stage. In cancer, the quantity of aneuploidy in a tumor correlated to its invasiveness. Whole-arm or whole-chromosome somatic copy number alterations (SCNAs) affect ca. 25% of the genome of a cancer cell, while focal SCNAs affect ca. 10% [82].

A tumor’s degrees of aneuploidy also correlate with its abilities to disarm a host’s immune responses against the invading tumors. The greater the aneuploidy, the less immune systems resist invading tumors [65]. Highest levels of aneuploidy in a malignancy (> 70th percentile of SCNA score) were associated with reduced T cell numbers but increased immune suppressive macrophages, thus controlling the tumor’s micro-environment and immune components independently of affecting selection of the T cell repertoire by neoantigen epitopes.

T cells attack tumors in various ways. One suppressive mechanism was recently identified in drug-resistant malignant gestational trophoblastic disease (where invasiveness of fetal tissue apparently does not shut off) [83]: As in other solid tumors [84], Pembrolizumab, a monoclonal antibody that blocks the tumor-expressed programmed cell death ligand 1 (PD-L1) signaling to the T cell inhibitory receptor (programmed death protein 1[PD-1]) pathway, was found clinically effective in inhibiting tumor growth.

In mice, PD-L1 expression has been demonstrated to maintain gestational tolerance, while loss of PD-L1 signaling resulted in fetal rejection [85]. Since PD-L1 is strongly expressed in trophoblastic malignancies, it is suspected to be involved in certain solid tumors’ immune-evasive properties [83]. Like tumor cells, blastocysts of early stage human embryos in general demonstrate excessive expression of gene products that favor cell progression, while not demonstrating expression of cell cycle check point genes [86].

Further strengthening the analogy, microenvironments of implanting embryos demonstrate on multiple levels remarkable similarities to microenvironments of malignant tumors. Exactly as seen in malignancies, TE aneuploidy may, thus, not only relate to invasiveness of early stage embryos (i.e., implantation capacity) but also to how embryos may affect local immune responses within microenvironments of implantation sites. Paradoxically, this would invert the current PGS/PGT-A hypothesis, as more aneuploid TEs might then denote better rather than poorer implantation chances.

Using whole transcriptome microarrays and enrichment analyses with GO gene sets, a recent mouse study, comparing global fetal tissues and tumor microenvironments, supported these conclusions: Central pathways toward tolerance induction in both clinical circumstances were remarkable similar in antigen-presentation, lymphocyte activation and T-regulator cell (Treg) activation [87].

## Implantation

Improved understanding of immunology and invasiveness of human embryo implantation also raises questions about the so-called (hormonally-determined) implantation window, defined by cycle days 20–24 (6–10 days after ovulation) [88, 89], a hypothesis requiring synchronization between endometrium and embryo development, with asynchrony of greater than 3.0 ± 1.5 days resulting in infertility [90].

To a degree, the high prevalence of extra-uterine pregnancies in humans, however, contradicts this hypothesis. So is also the previously noted observation that human embryos apparently developed normally up to post-fertilization day-14 in in vitro culture lacking endometrium and/or maternal contributions [49, 50]. That implantation depends on specific qualities of maternal endometrium, therefore, appear increasingly unlikely. More likely, once embryos enter the endometrial cavity, it is them who affect the endometrium.

In a mouse model, microenvironments in both uterine horns developed very differently depending on whether cleavage-stage or blastocyst-stage embryos were transferred into a uterine horn [91], suggesting that, likely “educated” by signals from “entrance-seeking” embryos, endometrial plasticity adjusts to developmental stages of embryos. Communications between embryos and endometrium during those 48 h of “floatation” in the endometrial cavity before implantation are also suggested by in vitro studies, suggesting an innate ability of interstitial endometrial cells to “sense” embryo quality, and favor good over bad [92, 93].

Combining immunology, invasiveness and here noted endometrial plasticity in response to embryonic guidance into a new all-encompassing implantation hypothesis, it appears that the endometrium, in fulfilling an innate immunobiological function of protecting women from bacteria, parasites, cancer cells and other potentially harmful invaders, is *always* “hostile” and, therefore, also “hostile” to implantation. This immunological hostility is only overcome, once invaders, like parasites [80] or semi-allogeneic embryos establish adequate systemic immunological tolerance, and uteri (or in cases of extra-uterine pregnancies other organs), therefore, become permissible to implantation.

Implantation, thus, appears driven mostly by biologically desirable embryos, with implantation sites being reactive, rather than proactive. Considering timing of entry of embryos into the endometrial cavity, adequate tolerance development usually, still, falls on cycle days 20–24 (6–10 days after ovulation), and “synchrony,” indeed, still appears to fall within 3.0 ± 1.5 days; those are, however, secondary effects and, because of endometrial plasticity, have limited clinical relevance. Primarily, implantation must be viewed as an immunologically- rather than endocrinologically-determined process.

An immunological definition of implantation clinically, however, demands new diagnostic capabilities of defining what represents adequate tolerance and, therapeutically, how tolerance pathways of pregnancy can be boosted when inappropriately low, and leading to implantation failure, miscarriages and/or late pregnancy complications, like premature labor [94].

## The ovarian cycle

Since introduction of gonadotropin therapy approximately six decades ago, female infertility treatments almost exclusively only concentrated on the last 2 weeks of follicle maturation, the so-called gonadotropin-dependent stages of folliculogenesis. Treatment was oriented toward the monthly menstrual cycle and, therefore, treatment cycles were defined by the approximately monthly interval between two menstrual periods.

Once recruited out of resting stage (primordial follicles), a follicle’s journey of maturation toward gonadotropin dependency takes at minimum two, and possibly as long as 4 months. Pharmacological interventions in the gonadotropin-dependent phase, therefore, occur relatively late, when follicle and oocyte quality (and quantity) to a large degree have already been determined by earlier events [95]. Further improvements, therefore, must come from interventions into earlier stages of follicle maturation, including manipulations of follicle recruitments out of the primordial follicle pools of resting follicles [96].

Such efforts have been under way for some time, as the mechanisms underlying follicle recruitment are increasingly well understood, with the mTor pathway apparently playing an important role [96]. Concomitantly, the importance of anti-Müllerian hormone (AMH) in restraining recruitment has been better elucidated [97].

**Fig. 1:**
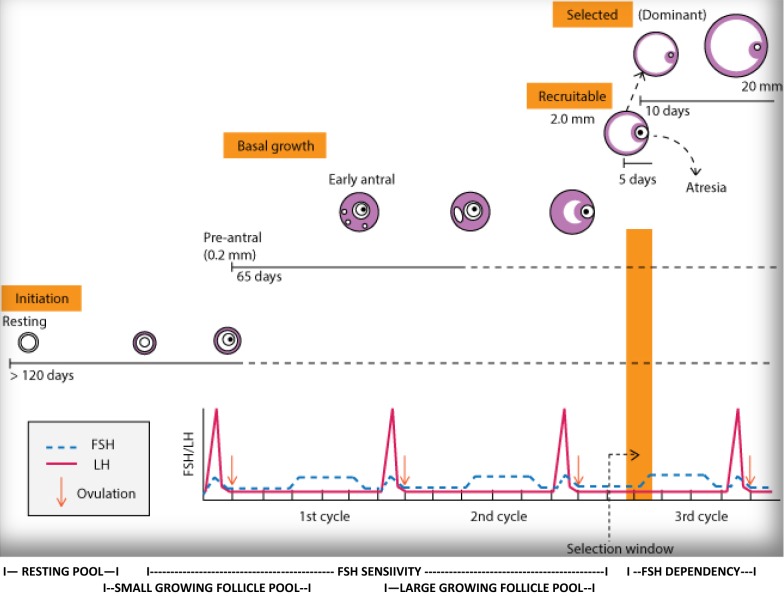
Folliculogenesis. The process of folliculogenesis, the time a single recruited follicle takes to reach ovulation (or retrieval in an IVF cycle, assuming it does not undergo atresia and apoptosis before) extends over multiple menstrual cycles. From a follicle’s vantage point, a real treatments cycle, therefore, involves multiple menstrual cycles. With therapeutic interventions, like androgen and HGH supplementations, moving into earlier stages of follicle maturation that the last 2 weeks of FSH-dependency, what is considered a treatment cycle, therefore, requires reevaluation

The utility of a “monthly” treatment cycle, therefore, becomes questionable, instead suggesting a new definition of what constitutes a treatment cycle as the time period it takes for follicles to mature between initial recruitment up to ovulation and/or retrieval in an IVF cycle (Fig. 1).

Some efforts along these lines have been underway for over a decade. For example, androgen supplementation in women with low ovarian reserve increased in popularity once importance of adequate testosterone levels at small growing follicle stages became apparent in enhancing FSH-sensitivity of granulosa cells [98]. This first example of earlier intervention into folliculogenesis also demonstrates the importance of ovarian microenvironments for follicle maturation and raised interesting questions about currently still widely held believes as to what constitutes ovarian aging.

As with advancing age female fecundity declines, ovaries are believed to age because of constant loss of finite follicle/egg numbers and declining (mostly chromosomal) quality of oocytes. Androgen supplementation reports, however, suggest that declining oocyte quantity and quality may not be consequence of only oocyte aging. That raising androgen levels in hypo-androgenic women improves ovarian function, pregnancy and live birth chances, instead suggests that reconstitution of aging ovarian microenvironments, in which follicles mature, may at least partially reverse ovarian aging. In other words, at least part of ovarian aging is consequence of aging of the ovarian micro-environment rather than of oocytes.

Difference between these two ovarian aging hypotheses are of great theoretical and practical importance because any physical damage from presumed aging of oocytes within primordial follicles must be considered irreversible; as androgen supplementation demonstrated, reconstitution of ovarian microenvironments with due to age insufficient components, is, however, possible. This new hypothesis of ovarian aging developed at New York’s *Center for Human Reproduction*, therefore, for the first time suggests that in some women quality of oocytes can be improved, while the traditional hypothesis of ovarian aging offers no such option.

Therapeutic interventions into earlier stages of folliculogenesis, however, also suggest a new definition for what constitutes an ovarian treatment cycle as the time between follicle recruitment and ovulation and/or oocyte retrieval in IVF cycles and, therefore, includes multiple menstrual cycles (Fig. 1).

Clinical practice increasingly points toward acceptance of this concept. Again, androgens are a good example: Since androgen supplementation primarily benefits small growing follicles [97], those follicles still require weeks–months to reach gonadotropin-dependency and ovulation. Androgen supplementation, therefore, must be initiated weeks–months before affected follicles/oocytes become available to gonadotropin stimulation and should be carried through till cycle completion [99]. A second example are recently reported new ovarian stimulation protocols, allowing stimulated cycle starts in either follicular or luteal phases, and even back to back double-stimulations in follicular and luteal phases [100, 101]. Both of these practice changes are based on the recognition that follicle maturation is a constant process; some follicles, therefore, reach gonadotropin dependency every day.

Going forward, such an updated definition of the ovarian cycle should also enhance ongoing efforts to learn to control this cycle in vitro.

## Conclusions

We here outlined some of the major developments we predict to enter clinical practice within the foreseeable future. Some will be truly groundbreaking—revolutionary, while others may appear more evolutionary.

Reviewing the modern history of fertility treatments, only three events can be characterized as truly groundbreaking: The introduction of gonadotropin therapy by Gemzell [102] and Lunenfeld [103], as already noted IVF [8] and, likely, the addition of intracytoplasmic sperm injection (ICSI) to IVF by Palermo et al. [104] in 1992, which offered genetic paternity to almost all male infertility patients. These three new treatments were groundbreaking because each of them allowed large new groups of previously infertile, and often sterile, couples to become parents. No other fertility treatments can make this claim.

Advancing female age, proportionally and progressively, has assumed ever larger swats of the fertility landscape. In developed countries, infertility in older women has in recent years become the quintessential infertility problem, as steadily improving IVF outcomes allow younger patients relatively quick conceptions, while older women often linger on in the system.

Older women will, however, be the primary beneficiaries of here discussed impending outcome improvements from translational clinical applications of recent research developments. As also noted, with ever older women conceiving, major societal changes will follow, for which medicine and society must appropriately prepare. Here described likely new developments now appear closer than even only a few years ago. Potential impacts on society, therefore, likely will occur sooner and at more rapid pace than previously anticipated.

Because the early embryo contains all the genetic information of a lifetime of health and disease, discoveries in reproductive biology often impact many other areas of human medicine. Developments in reproductive biology, therefore, will not only benefit human reproduction but all of medicine. It is for that reason that continuous uninhibited ethical research in reproductive biology and reproductive clinical medicine is of such crucial importance, if the U.S. is to maintain its leadership position in medical sciences.

## Abbreviations

BMP: bone morphogenic protein
CDC: Center for Disease Control
ESC: embryonic stem cell
FDA: Food and Drug Administration
GSC: germline stem cell
iPSC: induced pluripotent stem cell
IVF: in vitro fertilization
mDNA: mitochondrial DNA
nDNA: nuclear DNA
FGF: fibroblast growth factor
ICM: inner cell mass
ICSI: intracytoplasmic sperm injection
IND: investigational new drug
IRB: Institutional Review Board
OHSS: ovarian hyperstimulation syndrome
OR: ovarian reserve
PGD: preimplantation genetic diagnosis
PGS: preimplantation genetic screening
PGT-A: preimplantation genetic testing for aneuploidy
SCNA: somatic copy number alteration
TCL: trisomy-based chromosome loss
TE: trophectoderm
Treg cell: T regulator cell
